# Plant Hormone Homeostasis, Signaling, and Function during Adventitious Root Formation in Cuttings

**DOI:** 10.3389/fpls.2016.00381

**Published:** 2016-03-31

**Authors:** Uwe Druege, Philipp Franken, Mohammad R. Hajirezaei

**Affiliations:** ^1^Department Plant Propagation, Leibniz Institute of Vegetable and Ornamental CropsErfurt, Germany; ^2^Department of Molecular Plant Nutrition, Leibniz Institute of Plant Genetics and Crop Plant ResearchGatersleben, Germany

**Keywords:** adventitious rooting, plant development, cutting, wound, hormones, PIN, signaling, cell fate

## Abstract

Adventitious root (AR) formation in cuttings is a multiphase developmental process, resulting from wounding at the cutting site and isolation from the resource and signal network of the whole plant. Though, promotive effects of auxins are widely used for clonal plant propagation, the regulation and function of plant hormones and their intricate signaling networks during AR formation in cuttings are poorly understood. In this focused review, we discuss our recent publications on the involvement of polar auxin transport (PAT) and transcriptional regulation of auxin and ethylene action during AR formation in petunia cuttings in a broad context. Integrating new findings on cuttings of other plant species and general models on plant hormone networks, a model on the regulation and function of auxin, ethylene, and jasmonate in AR formation of cuttings is presented. PAT and cutting off from the basipetal auxin drain are considered as initial principles generating early accumulation of IAA in the rooting zone. This is expected to trigger a self-regulatory process of auxin canalization and maximization to responding target cells, there inducing the program of AR formation. Regulation of auxin homeostasis via auxin influx and efflux carriers, GH3 proteins and peroxidases, of flavonoid metabolism, and of auxin signaling via AUX/IAA proteins, TOPLESS, ARFs, and SAUR-like proteins are postulated as key processes determining the different phases of AR formation. NO and H_2_O_2_ mediate auxin signaling via the cGMP and MAPK cascades. Transcription factors of the GRAS-, AP2/ERF-, and WOX-families link auxin signaling to cell fate specification. Cyclin-mediated governing of the cell cycle, modifications of sugar metabolism and microtubule and cell wall remodeling are considered as important implementation processes of auxin function. Induced by the initial wounding and other abiotic stress factors, up-regulation of ethylene biosynthesis, and signaling via ERFs and early accumulation of jasmonic acid stimulate AR formation, while both pathways are linked to auxin. Future research on the function of candidate genes should consider their tissue-specific role and regulation by environmental factors. Furthermore, the whole cutting should be regarded as a system of physiological units with diverse functions specifically responding to the environment and determining the rooting response.

## Introduction

Adventitious root (AR) formation is a developmental process, where new roots are generated spontaneously or in response to certain stimuli from stems, leaves, or non-pericycle tissues of older roots (Li et al., 2009). Thus, ARs are formed from cells of non-root pericycle identity, which have to be newly determined to start a root developmental program. On the one hand, this process presents a model for the enormous plasticity of plants. On the other hand, it provides the basis for clonal multiplication, a technology which is utilized for breeding and production of a great proportion of crop and forestry plants. AR formation is a multiphase process and particularly observed in excised plant parts (cuttings), where it is the combined response to two stimulating principles (**Key Concept 1**).

KEY CONCEPT 1 **Excision-induced AR formation** involves two stimulating principles: wounding at the cutting site and isolation from the resource and signal network of the whole plant. In dependence on the plant genotype and physiological condition, ARs develop in non-root tissues of the excised plant parts in response to these two stimuli or require an extra stimulus such as auxin application.

**Figure 1:**
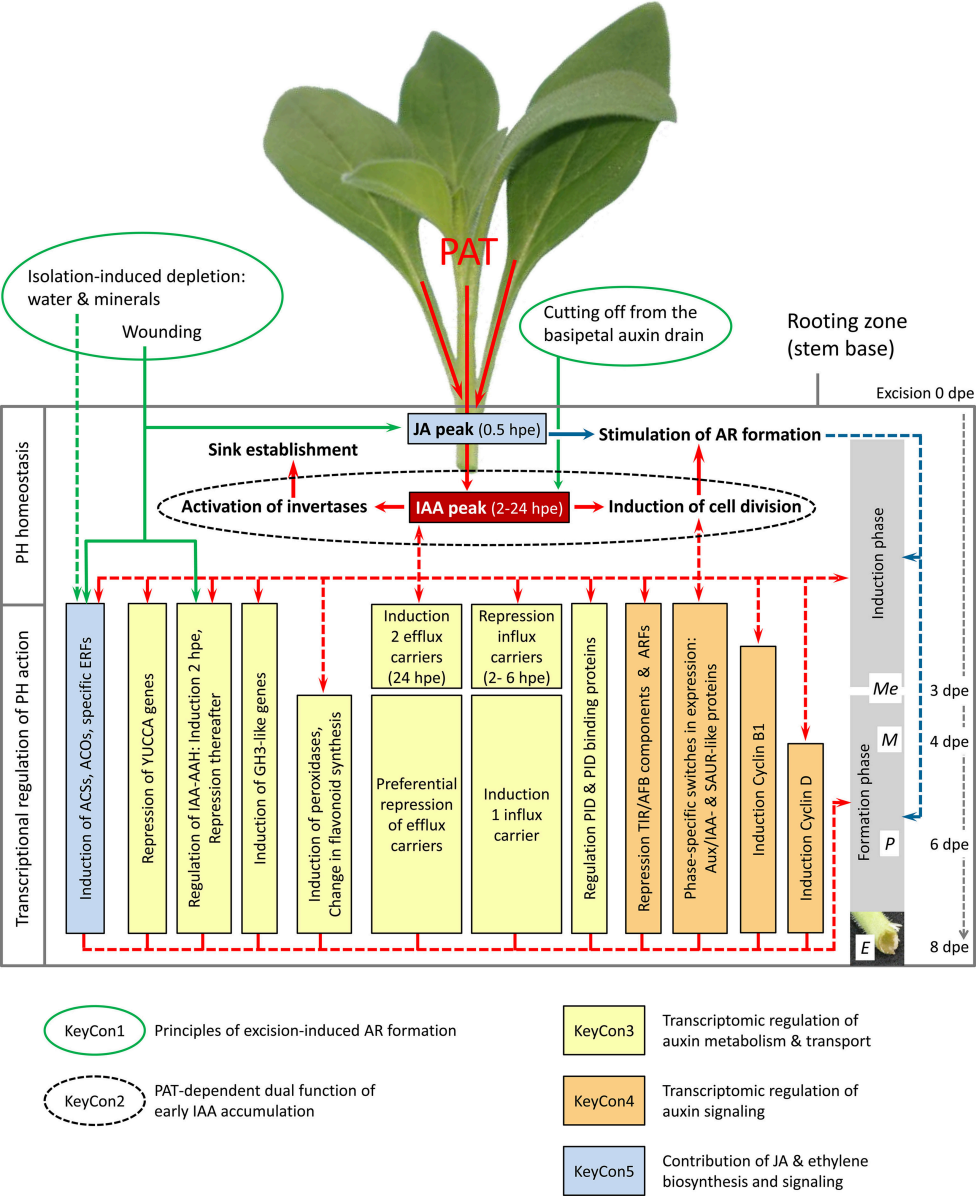
**Network of ethylene, auxin and jasmonic acid homeostasis and signaling in excision-induced AR formation in *Petunia hybrida* cuttings**. Factors underlying the **Key Concepts 1–5** are indicated by specific framing and colors. Green arrows indicate evident and hypothetic (dashed line) factors stimulating IAA and JA accumulation and inducing specific *ACS, ACO*, and *IAA-AAH* genes in the rooting zone. Red arrows indicate evident links between PAT, IAA accumulation, invertase activity, cell division and AR formation and hypothetical links (dashed arrows) between the resulting IAA level, transcriptional regulation of plant hormone (PH) action and the two phases of AR formation. Blue arrows indicate the evident link between early JA accumulation and AR formation, while the action on induction and/or formation is still unclear (dashed arrows). PID, PINOID; Me, meristemoids; M, meristems; P, primordia. The scheme integrates the two models of Ahkami et al. (2013) and Druege et al. (2014) and recent results of Lischewski et al. (2015).

On an anatomical scale, AR formation starts with the induction phase, which is devoid of any visible cell divisions, but involves the reprograming of target cells toward the establishment of clusters of new meristematic cells (root meristemoids). The induction phase can be further separated in the early and late phase (da Costa et al., 2013), while the early induction phase may include the dedifferentiation of founder cells as postulated by De Klerk et al. (1999). The induction phase is successively followed by the formation of the dome-shaped root primordia (initiation phase) and by the establishment of vascular connections of the new structures and root emergence (expression phase). For simplification purposes, the initiation and expression phases can be joined under the single domination of formation phase. Whereas ARs in young hypocotyls originate from pericycle cells, ARs in older hypocotyls, non-hypocotyl stems and petioles of detached leaves are initiated in other tissues in close proximity to the vascular tissues, such as phloem or xylem parenchyma cells, or interfascicular cambium cells (da Costa et al., 2013; Bellini et al., 2014). For clonal propagation, the excised leafy tip of an axillary shoot of the donor plant is the structure frequently used as cutting, where the ARs are generated in the non-hypocotyl stem base (Figure 1).

Plant hormones play an important role in the control of AR formation as they respond to the changing environment, provide a signaling network within the plant and are decisive for cell fate determination and specification. However, the knowledge of the control and function of plant hormone homeostasis and of the intricate signaling network during AR formation in cuttings is fragmentary. In two recent comprehensive reviews, da Costa et al. (2013) and Pacurar et al. (2014) discussed the knowledge on the function of plant hormones in AR formation with special emphasis on the role of stress and on auxin as master regulator of AR formation as central player in a hormonal network. Even though both reviews considered the particular situation of cuttings when compared to intact plants, the concepts on the function of plant hormones in AR formation in cuttings are widely based on results obtained in other systems such as intact hypocotyls particularly of *Arabidopsis* and on the much more abundant knowledge on lateral root development.

In this focused review, particular attention is given to recent findings related to the hormonal control of AR formation in cuttings. Based on our recent publications on the involvement of polar auxin transport (PAT) and transcriptional regulation of hormone-related pathways during AR formation in petunia cuttings (Ahkami et al., 2013; Druege et al., 2014), we discuss new related findings on cuttings and explants also of other plant species while further considering current general models on plant hormone networks. Taking into account, that AR formation in non-hypocotyl and hypocotyl cuttings of *Arabidopsis*, despite the different founder tissues, involve similar transcriptional regulation (Welander et al., 2014) new findings on hypocotyls and thin cell layers (TCLs) of *Arabidopsis* are included. A general model on the regulation and function of auxin, ethylene, and jasmonate action during AR formation of cuttings is developed and future challenges to improve the understanding of these relationships are discussed.

## Involvement of auxin and ethylene homeostasis and signaling in excision-induced AR formation in petunia

It is widely accepted that auxins, mainly indole-3-acetic acid (IAA) is an effective inducer of AR formation, whereas high auxin levels obviously have an inhibitory role at later stages (De Klerk et al., 1999; Ludwig-Müller, 2009). Auxin application has also been found to increase the translocation of assimilates from the leaves and the sugar abundance at the site of root development (Agullo-Anton et al., 2011 and references therein). Though, experimental data support the view that excision-induced AR formation in cuttings is dependent on PAT and involves early accumulation of IAA in the rooting zone (Blazkova et al., 1997; Tonon et al., 2001; Garrido et al., 2002), a coherent picture on the functional relationship between PAT, auxin accumulation, and induction of AR formation was lacking until recently. We used *Petunia hybrida* to analyze the relationships between plant hormones and excision-induced AR formation in cuttings. First, we studied the role of PAT in the temporal distribution of IAA in the rooting zone and relationships to carbohydrate metabolism and AR formation (Ahkami et al., 2013). In another approach, we used a microarray to analyze the transcriptome of plant hormone-related genes in the stem base during excision-induced AR formation (Druege et al., 2014). The most relevant results and our current concepts on the involvement of hormone-related regulation of AR formation are discussed in the following chapter and illustrated in Figure 1.

Petunia cuttings excised and planted for rooting exhibited a double peak of IAA in the stem base between 2 and 24 h post excision (hpe). However, blocking of PAT with naphthylphthalamic acid (NPA) completely prevented the 24-hpe peak of IAA and severely inhibited the root meristemoid formation and final rooting of cuttings. Furthermore, NPA treatment reduced the activities of vacuolar and cell wall invertases, which are considered as molecular drivers of sink activity in the newly developing sink of the rooting zone (Ahkami et al., 2009), during the early induction phase. The results provide evidence that early accumulation of IAA in the rooting zone of petunia is dependent on PAT and has a dual function in AR formation (**Key Concept 2**, Figure 1).

KEY CONCEPT 2 **PAT-dependent early accumulation of IAA in the rooting zone has a dual function:** Induction of AR formation in responding target cells via control of cell cycle involving cyclins and via contribution to sink establishment in the rooting zone involving sucrolytic enzymes.

Complementing the finding that the IAA-peak at 24 hpe was highly dependent on PAT, the transcriptome did not reveal any indication for excision-induced up-regulation of genes involved in auxin biosynthesis. By contrast, one gene encoding a flavinmonooxygenase of the YUCCA family, the bottleneck of tryptophan-dependent IAA biosynthesis was repressed throughout the rooting period. However, early up-regulation of two isogenes of IAA-amino acid hydrolase (IAA-AAH), being also wound-inducible in leaves, may contribute to the very early IAA peak at 2 hpe via hydrolysis of amino acid conjugates, whereas the subsequent repression of the same genes may contribute to the observed reduction of IAA level during the root formation phase (Figure 1). Seven out of eleven genes encoding proteins of the GH3 family showed a prolonged induction, while six transcripts exhibited a strong induction at 2 hpe already. One major function of GH3 proteins is their activity as IAA-amido synthetases, which are important for maintaining auxin homeostasis via conjugation of IAA to amino acids (Staswick et al., 2005). The data suggest that shortly after excision the auxin metabolism is shifted toward long-term reduction of the IAA pool (**Key Concept 3**). Considering that in intact hypocotyls of *Arabidopsis* one particular GH3 protein acts as positive regulator of de-etiolation-induced AR formation via conjugation of inhibitory JA (Gutierrez et al., 2012), some of the upregulated *GH3* genes in petunia may have other functions beyond conjugation of IAA. Taking into account the catalytic activity of peroxidases on IAA, the long-term reduction of the IAA pool is probably further supported by the observed pronounced increase in the expression of peroxidases from 72 hpe onwards (Figure 1).

KEY CONCEPT 3 **AR formation in cuttings involves a transcriptomic shift toward long-term reduction of the IAA pool and a fine-tuning of the auxin transport machinery**, regulating auxin canalization toward and maximization in particular target cells.

Regulation of PAT involves specific transporters and/or channels for influx [(auxin permease (AUX), like AUX (LAX)], and efflux [pin-formed (PIN)], and the multidrug resistance/P-glycoprotein auxin carriers [MDR/PGP] as sub-family of ATP-binding cassette (ABC) transporters. Turnover, cycling and trafficking of these proteins contributes to the asymmetric and dynamic nature of PAT (Kerr and Bennett, 2007; Morris et al., 2010). Even though AR formation in petunia shows a strong dependency on PAT, the microarray data does not indicate a general stimulation of the auxin transport machinery but rather points to a phase-specific fine-tuning of the system at transcriptional level (Figure 1, **Key Concept 3**).

One gene for a putative efflux transporter was up-regulated from 2 hpe onwards and reached a 20-fold increase at 24 hpe when one PIN-like gene for an auxin transport protein was induced too. Up-regulation of these efflux carrier genes (Figure 1) may have contributed to the observed PAT-dependent auxin peak at 24 hpe and to the induction of AR formation. However, considering that three of four regulated efflux carrier genes were down-regulated before 24 hpe, we assume that auxin accumulation at least partially reflects a non-transcriptionally regulated overflow of auxin resulting from the interruption of the basipetal auxin drain after excision of the cuttings. This view is supported by auxin transport data from *Arabidopsis* and pea indicating that in stems there is considerable excess capacity to transport widely varying amounts of auxin (Renton et al., 2012). In petunia cuttings, two out of six influx carrier genes were down-regulated during the induction phase. However, one of them was continuously up-regulated during the root formation phase, when four out of six efflux transporter genes were downregulated. The data suggest a preferential role of auxin influx carriers during the formation of new meristems and subsequent differentiation. This stays in line with the proposed function of AUX/LAX controlled, acropetal auxin flux to the root apex as important factor controlling embryonic and lateral root development (Swarup et al., 2001; Peer et al., 2011). In addition, the petunia array data indicate differential expression of the protein kinase PINOID (Figure 1) targeting PINs to the apical plasma membrane (Fozard et al., 2013) and of PINOID-binding proteins which modify PINOID activity (Benjamins et al., 2003). This suggests a further fine-tuning of the auxin transport machinery via intracellular localization of carriers. Flavonoids modify auxin transport in dependence on their quality particularly by interaction with efflux carriers (Peer and Murphy, 2007; Santelia et al., 2008). Therefore, the observed shift in expression of genes of the flavonoid pathway (Figure 1) can be expected to modify auxin transport. Considering the strong feedback between auxin level and regulation of auxin carriers (Habets and Offringa, 2014), we hypothesize that the differential expression of auxin carriers and their controlling kinases is partially auxin-induced as the result of the initial auxin overflow. We postulate that the early accumulation of auxin in the rooting zone as a result of ongoing PAT and cutting off from the basipetal auxin drain initiates self-regulatory auxin canalization toward and concentration increase (maximization) in particular target cells (Bennett et al., 2014), there starting the program of AR formation (**Key Concept 3**). The important role of auxin allocation to particular cells as principle of AR induction is supported by studies of Sukumar et al. (2013) on de-rooted *Arabidopsis* seedlings of a *pGH3-2:GUS* reporter line, where auxin maxima were detected in pericycle cells, the sites for subsequent root primordia formation. Considering that in petunia also 76, 75, 32, and 18 genes putatively controlling “protein synthesis, processing and degradation,” “gene expression and RNA metabolism,” “membrane transport,” and “vesicular trafficking, secretion and protein sorting” were up-regulated during the induction phase (Ahkami et al., 2014), it can be expected that beyond the transcriptional regulation of auxin homeostasis post-transcriptional processes should also be involved.

The control of AR formation at the level of auxin signaling is poorly understood. A current model on the involved signaling cascade is based on studies on primary and lateral root development and on other developmental processes (da Costa et al., 2013). Important regulating components in the nucleus are the TIR1/AFB complex (transport inhibitor response/auxin-signaling F-box) and the repressor proteins Aux/IAA. The latter recruit the co-repressor TPL (TOPLESS), being also a co-repressor in jasmonate signaling, to exert their repressive function on ARFs (auxin response factors; Chapman and Estelle, 2009; Perez and Goossens, 2013). IAA cross-links the TIR1/AFB complex and the repressor proteins Aux/IAA. This allows ubiquination and proteosomal degradation of the repressor Aux/IAA, releasing the ARFs from repression. The released ARFs act as activators or repressors on the transcription of auxin-responsive genes. In *Arabidopsis, ARF6* and *ARF8* have been identified as positive and *ARF17* as negative regulators of de-etiolation-induced AR formation in intact hypocotyls (Gutierrez et al., 2012). In petunia cuttings, many genes putatively encoding ubiquitin-ligases of the TIR/AFB complex and ARFs were down-regulated shortly after excision, whereas up-regulation was only rarely observed (Figure 1). This response reflects a transcriptomic response toward overall reduction of auxin sensitivity after cutting excision (**Key Concept 4**), which may be based on a negative feedback (Benjamins and Scheres, 2008) to the early rise in IAA level. Because certain ARFs may act as repressors of AR formation (Gutierrez et al., 2012), the observed early down-regulation of certain ARFs may have contributed to the induction of AR formation.

KEY CONCEPT 4 **AR formation in cuttings involves a strong transcriptional modification of the auxin signaling cascade.** Early repression of components of the TIR/AFB complex and ARFs indicate desensitizing to auxin, while differential expression of Aux/IAA genes and certain genes for ARFs and SAURs along the phases indicate phase-specific functions.

Genes putatively coding for Aux/IAA proteins showed a strong regulation and the most phase-specific shifts in expression (Figure 1), which were mainly pronounced between 6 and 72 hpe. ARFs and Aux/IAAs have already been considered as important “auxin codes” for reprogramming phases of other developmental processes (Teale et al., 2006). The strong temporal variation in Aux/IAA expression supports the view, that these are important selective controllers of auxin response pathways during the specific phases of AR formation (**Key Concept 4**). Because the expression of Aux/IAA proteins is highly sensitive to auxin (Benjamins and Scheres, 2008) and also provides a linkage to other plant hormones (Brenner et al., 2005; Song et al., 2009; Çakir et al., 2013), the transcriptional regulation can be expected to reflect both the changes in IAA level and crosstalk to other hormones during AR formation. SMALL AUXIN UP RNAs (SAURs) are genes, of which several have been shown to be transcriptionally induced by auxin in diverse plant species (Ren and Gray, 2015), but the knowledge on their role in root development is fragmentary. Whereas in rice overexpression of one SAUR isogene reduced root elongation and lateral root development (Kant et al., 2009), overexpression of two other isogenes in *Arabidopsis* increased elongation of the primary root (Markakis et al., 2013) and the number of lateral roots (Kong et al., 2013). Interestingly, in petunia, genes coding for SAUR-like proteins were strongly regulated after excision of the cuttings (Figure 1) and, the strongest shift in expression was observed between 6 and 3 dpe (days post excision). This suggests that specific SAUR-like proteins have also particular functions in the induction and differentiation in excision-induced AR formation (**Key Concept 4**). To date no molecular or biochemical mechanism has been elucidated to explain how SAUR proteins might regulate root development (Ren and Gray, 2015). However, considering that in the shoot SAURs probably control cell expansion via targeting PP2C.D phosphatases which act as inhibitors of plasma membrane H^+^-ATPases (Ren and Gray, 2015), a similar function may also be involved in AR formation. Furthermore, similar to the Aux/IAA proteins, SAUR proteins are regulated also by other hormones than auxin including ethylene and jasmonic acid (Nemhauser et al., 2006; Ren and Gray, 2015; Li et al., 2015a) providing a possible linkage point for hormonal crosstalk during AR formation.

Cyclin-dependent kinases and cyclins as their regulatory proteins are involved in auxin- and cytokinin-mediated governing of the cell cycle (Hartig and Beck, 2006; Komaki and Sugimoto, 2012). Expression of cyclin genes has already been related to auxin-induced AR formation in *Pinus* and *Quercus* (Lindroth et al., 2001; Neves et al., 2006). In petunia cuttings, transcripts coding for Cyclin B1 and Cyclin D accumulated from 2 and 4 dpe onwards in the stem base, respectively (Ahkami et al., 2009, 2014). Further considering, that in the pericycle of *Arabidopsis* hypocotyl cuttings *pCYCB1:GUS* expression was detected after the *pGH3-2:GUS* expression (Sukumar et al., 2013), we propose the transcription of cyclin genes as important regulative factor mediating the auxin control of AR formation in cuttings via the cell cycle (**Key Concept 2**, Figure 1).

Ethylene, which biosynthesis is strongly enhanced by injury and diverse other stresses (Druege, 2006), has recently been considered as stimulant of early induction and late formation (expression phase) of ARs but inhibitory during the late induction phase, while generally strongly interlinked with auxin (da Costa et al., 2013). Transcriptome data of petunia AR formation suggest a strong excision-induced stimulation of ethylene biosynthesis at the levels of ACC (aminocyclopropane) synthesis and oxidation (Figure 1). While a substantially high number of genes of ACC synthase (ACS) and ACC oxidase (ACO) were also induced in leaves within 2 h after wounding, most of the genes were continuously up-regulated during AR formation in the stem base. However, the time points of maximal induction indicated different principles of stimulation. We consider wounding and deficiency in water and in minerals in response to the separation from the root system as important environmental factors and the IAA accumulation during the induction phase as hormonal factor stimulating the ethylene biosynthetic pathway. Our finding that applications of AVG, an inhibitor of ACS activity, and of ACC to de-rooted seedlings reduced and enhanced the number of ARs, respectively, clearly demonstrates that enhancing ethylene biosynthesis within a physiological range has a positive effect on the number of formed ARs. However, the observed inhibitory effect of ACC on average root length suggests that high ethylene levels reduce AR elongation. This is in agreement with findings in ARs and lateral roots of other plant species (Riov and Yang, 1989; Negi et al., 2008). The effect of high ethylene concentrations may be indirect via stimulating biosynthesis and transport of auxin into the elongation zone, where it inhibits root elongation (Muday et al., 2012). Many ethylene responsive transcription factors (ERFs) were continuously up-regulated during AR formation and also induced in wounded leaves (Figure 1). ERFs regulate ethylene-responsive genes and are considered to affect developmental processes, while their expression responds to hormones including ethylene and to many different external stimuli as e.g., abiotic stress factors (Ohme-Takagi et al., 2000; Licausi et al., 2013). These results and the finding that application of an inhibitor of ethylene perception strongly reduced AR formation in the cuttings, demonstrate the important role of ethylene signaling during AR formation (**Key Concept 5**). However, considering the plenty of regulated ERFs and also ethylene biosynthetic genes without marking specific time points, it appears that unlike the obvious master control by auxin, ethylene seems to be important to stimulate AR development but not to control the process *per se*. The positive regulatory role of ethylene in AR formation of cuttings is further supported by recent studies in other plant species. Compared to a weak rooting cultivar, a good rooting cultivar of carnation accumulated higher levels of ACC at 24 and 54 h after planting (hAP) and, ACC levels could be enhanced by applying auxin to stimulate AR formation (Villacorta-Martin et al., 2015). Liu et al. (2013) monitored a strong increase of ethylene evolution from the stem base of chrysanthemum cuttings peaking at 1 dpe. Furthermore, application of AgNO3 as inhibitor of ethylene perception before planting the cuttings inhibited AR formation.

KEY CONCEPT 5 **AR formation in cuttings is stimulated by ethylene production, ERF-mediated signaling and early jasmonate accumulation**.

## Role of JASmonate in AR formation in petunia and pea cuttings

**Figure 2:**
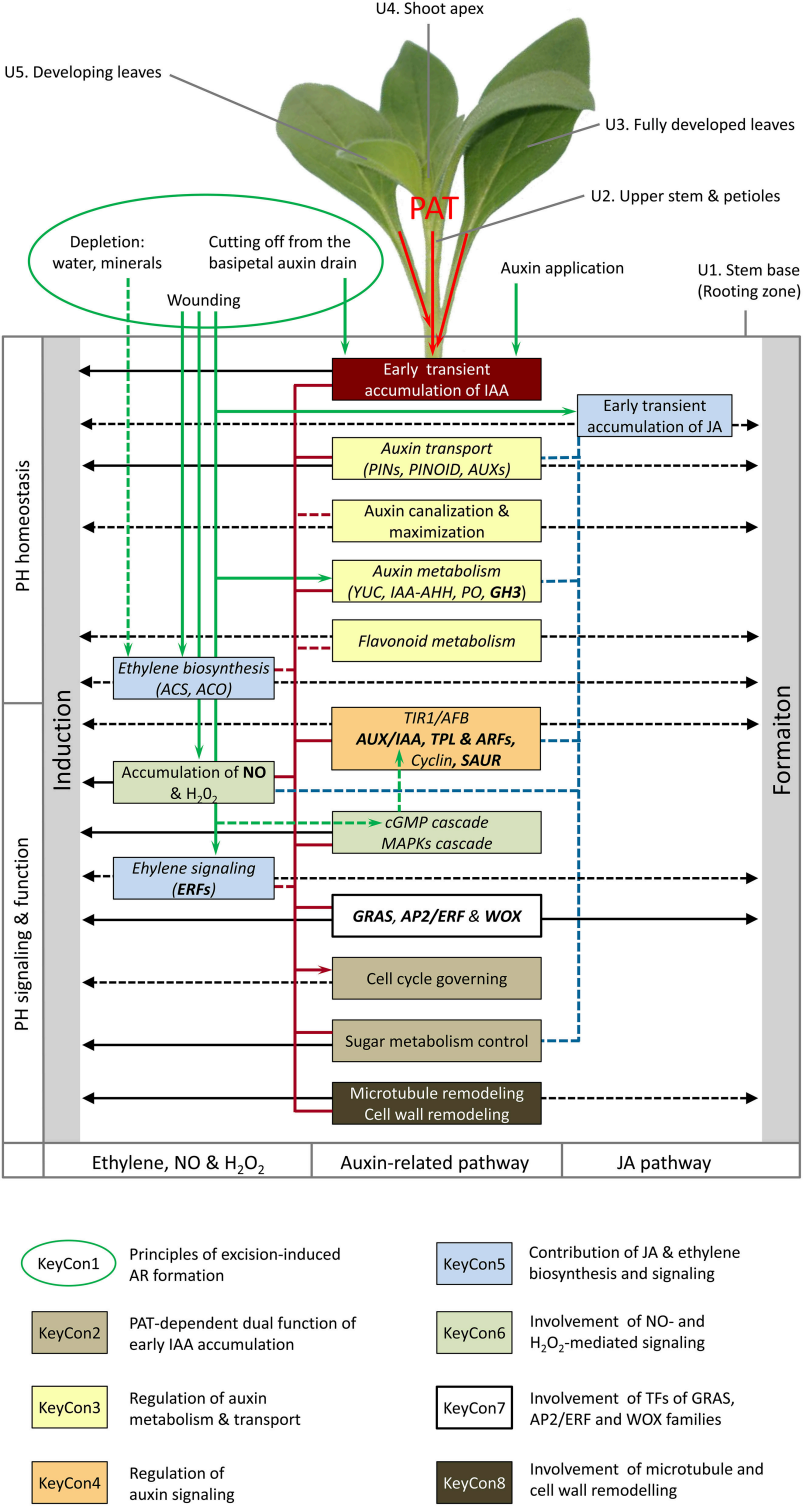
**General model of important physiological units of shoot tip cuttings and regulative factors controlling ethylene, auxin, and jasmonic acid homeostasis, signaling and function in AR formation**. Factors underlying the **Key Concepts 1–8** are indicated by specific framing and colors. Components with postulated phase-specific regulative character and crossroad functions between different plant hormones (PH) are indicated by italic and bold letters, respectively. Black arrows indicate evident or hypothetic (dashed lines) functions in induction and formation of ARs. Green arrows indicate evident (supported by data on cuttings) and hypothetic (supported by other data, dashed lines) factors stimulating accumulation of IAA (PAT-dependent), JA and NO, auxin biosynthesis and mobilization, and ethylene biosynthesis and signaling. Red lines indicate evident and hypothetic (dashed lines) linkages between components of ethylene and auxin biosynthesis, signaling and function. Blue dashed lines indicate linkages between JA and auxin homeostasis and signaling and invertase activation. Function of units (not complete): U1, rooting zone; U2, transport route of hormones and others; U3, carbohydrate source, potential source of auxin, U4, carbohydrate sink competing to the rooting zone (Klopotek et al., 2016), potential source of auxin; U5, carbohydrate sink, potential source of auxin. The scheme integrates the petunia model of Figure 1 and recent results obtained on other plant species, which are discussed in the text.

In the majority of plant species, wounding also leads to an increase in jasmonates (Schilmiller and Howe, 2005). In the stem base of petunia cuttings, Ahkami et al. (2009) found a strong accumulation of jasmonic acid (JA) during the induction phase, peaking at 0.5 hpe already, while the transient accumulation preceded the RNA accumulation of a member of the cell wall invertase gene family and the rise in the corresponding enzymatic activity. Based on these results, Lischewski et al. (2015) recently tested the function of JA in AR formation in petunia cuttings. A strong reduction of transcripts and activity of petunia allene oxide synthase, the rate limiting enzyme in JA biosynthesis, significantly reduced the levels of JA and its bioactive conjugate (+)-7-iso-jasmonyl isoleucine in the cuttings. This reduced the numbers of root primordia formed at 7 dpe and the number of ARs determined at 21 dpe confirming the positive role of JA in AR formation of petunia. Further analysis of hormone levels, cell wall invertase activity and related transcripts at time points of expected maxima did not indicate that JA functioning during AR formation is mediated via auxin homeostasis, ethylene biosynthesis or carbohydrate metabolism. The authors concluded that JA might act as an accelerator of AR formation (Figure 1). According to these results, Rasmussen et al. (2015) recently showed that AR formation in rooting-competent vegetative cuttings of pea exhibited an early rise in JA during the induction phase. This rise was delayed in low-rooting cuttings, which apical meristem had switched to floral identity. Interestingly AR formation in the rooting-competent cuttings could be enhanced by a pulse treatment with JA during the first 6 hpe. Considering also the positive effects of JA observed on AR formation in potato cuttings (Ravnikar et al., 1992), it appears that JA has a stimulating function in excision-induced AR formation particularly during the induction phase (**Key Concept 5**). This is in contrast to the negative role of JA in de-etiolation-induced AR formation in intact hypocotyls of *Arabidopsis* (Gutierrez et al., 2012). The particular roles of JA during the other phases of AR are, however, still in question (Figures 1, 2). Even though the auxin maximum in the stem base of petunia was not altered by inhibited JA biosynthesis (Lischewski et al., 2015), interrelationships between JA and auxin homeostasis and signaling, rarely understood yet (Perez and Goossens, 2013), should be further considered in future studies.

## Auxin homeostasis, signaling and function in other plant species

New findings on cuttings and explants from other plant species further support the important role of auxin homeostasis, signaling and function in AR formation in cuttings (**Key Concepts 2–4**). High rooting capacity of vegetative pea cuttings was associated with an early peak of IAA in the stem base, which was absent in low rooting cuttings with a floral apical shoot meristem (Rasmussen et al., 2015). Sukumar et al. (2013) showed that localized induction of the ABC B19 auxin transporter contributes to excision-induced AR formation in *Arabidopsis* hypocotyls while mutations of *ABC B19, PIN1, PIN3-3*, and *PIN7* genes produced significant reductions in AR formation. Li et al. (2015b) used RNA sequencing (RNA-seq) and *q*PCR to analyze the transcriptome in the hypocotyl of de-rooted seedlings of mung bean. Almost a 100 genes related to cell cycle were up-regulated at 24 hpe which was 24 h before AR primordia were formed and 72 h before ARs emerged. During induction (6 hpe), one gene encoding an ABC transporter G family member 22-like protein with a putative auxin efflux or influx carrier function was strongly down-regulated. A *PIN1* homolog was up-regulated during the induction and initiation (24 hpe) phase whereas a putative auxin influx transporter gene (*LAX4*) and seven genes encoding putative ARFs showed opposite responses. In addition, differential phase-dependent expression of eight auxin-responsive protein genes of the AUX- and IAA-type was observed. Villacorta-Martin et al. (2015) combined hormone analysis with RNA-seq-based monitoring of the transcriptome in the stem base of cuttings of two carnation cultivars with contrasting efficiencies of AR formation. After 8 h of cold dark storage, excised cuttings were treated either with synthetic auxins or a mock solution over the period of 15 h before planting. At 0 hAP, which was 23 hpe, a high IAA level was recorded only in the stem base of the good rooting cultivar, which, however, was strongly reduced by auxin application. The auxin treatment accelerated the activation of cell division and caused a higher number of root initials within the cambium of both cultivars. Because the poor rooting cultivar exhibited higher levels of trans-zeatin between 0 until 54 hPA and considering the inhibitory role of cytokinins on AR induction (da Costa et al., 2013), Villacorta-Martin et al. (2015) concluded that the weak rooting was related to the low ratio of auxin vs. cytokinin levels. Interestingly, several genes encoding regulators of auxin response were also found differentially regulated in carnation during the early phase of AR formation (0–6 hAP) before first cell divisions were recorded. Several genes encoding putative Aux/IAA repressors were specifically up-regulated at 6 hAP in the good rooting cultivar and were transiently induced by auxin application in both cultivars. Among them were three putative homologs of *SHY2* (also known as *IAA3*), *MASSUGU2* (*IAA19*), and *SOLITARY ROOT* (*IAA14*) in *Arabidopsis*. Further, several genes encoding sucrose degradation enzymes including four genes putatively encoding two vacuolar and two cell well invertases were up-regulated during the induction phase. Furthermore, a number of genes encoding mitotic A-type and B-type cyclins were up-regulated in both cultivars from 24 hAP onwards and their expression was enhanced by auxin application. These results and those of earlier studies on the role of PAT, auxin transporters and the response of carbohydrate metabolism in carnation cuttings (Garrido et al., 2002; Oliveros-Valenzuela et al., 2008; Agullo-Anton et al., 2011, 2014), further support the concept that in shoot tip cuttings PAT is decisive to provide early and transient accumulation of IAA in the rooting zone, where it induces AR formation by initiating a cascade of transcriptional responses related to auxin signaling and down-stream processes, e.g., cell division and carbohydrate sink establishment (Figure 2).

A recent study of de Almeida et al. (2015) provided new insights into the involvement of tissue-specific hormone homeostasis and signaling in rooting recalcitrance of *Eucalyptus globulus* cuttings when compared with non-recalcitrant *E. grandis.* Application of IAA during the induction phase to a great part rescued AR formation in the recalcitrant species. Auxin immunolocalization revealed that *E. grandis* accumulated more auxin at 48 hpe in the cambium zone as founder tissue of ARs than *E. globulus*, while IAA application enhanced auxin to similar levels in both species. Analysis of cambium specific gene expression by use of laser capture microdissection pointed the attention to the auxin-co-repressor gene *TPL*, the Aux/IAA-protein encoding gene *IAA12/Bodenlos* and the gene *ARR1*, a homolog of the cytokinin type-B response regulator *PtRR13* which exhibits a negative regulatory role in AR formation in poplar (Ramirez-Carvajal et al., 2009). All three genes were higher expressed in *E. globulus* 24–48 hpe and repressed by auxin treatment. These results strongly support the importance of local auxin accumulation (**Key Concept 3**) and of auxin-induced down-regulation of certain negative regulators such as Aux/IAA proteins, TPL (Figure 2), and cytokinin response regulators during induction of ARs in cuttings (**Key Concept 4**).

Numerous experimental evidences support the important role of nitric oxide (NO) as positive regulatory component of auxin signaling during AR formation in cuttings (reviewed in da Costa et al., 2013; Simontacchi et al., 2013). Interestingly, hydrogen peroxide (H_2_O_2_) can have a similar function independent or in combination with NO (Liao et al., 2009; Li and Xue, 2010) (**Key Concept 6**). Production of both compounds can be stimulated by wounding (Garces et al., 2001; Jih et al., 2003) and by auxin while accumulation during AR formation was shown to depend on PAT (Liao et al., 2011; Yadav et al., 2011). Impaired NO accumulation resulting from reduced expression of nitrate reductase is obviously involved in the maturation-mediated decline of AR formation in cuttings *of Eucalyptus grandis* (Abu-Abied et al., 2012). The explicit functions of NO and H_2_O_2_ in AR induction and formation and the linkage to the other signaling pathways controlling AR formation are far from being understood. However, Terrile et al. (2012) provided data supporting the concept that NO enhances auxin signaling via S-nitrosylation of the auxin receptor protein TIR1, thereby facilitating Aux/IAA degradation. Obviously depending on the plant, NO and H_2_O_2_ can modify AR formation via the calcium (Ca^2+^) cyclic guanosine monophosphate (cGMP) cascade and independent on cGMP via the mitogen-activated protein kinase (MAPK) cascade (Pagnussat et al., 2003, 2004; Lanteri et al., 2006; Liao et al., 2009, 2012; Li and Xue, 2010). Interestingly, cGMP has recently been shown to rapidly increase by wounding (Van Damme et al., 2014). Further, Nan et al. (2014) highlighted the involvement of cGMP in auxin-mediated primary root development and growth in *Arabidopsis*. Application of a cell-permeable cGMP analog enhanced auxin-induced expression of genes encoding Aux/IAA-, SAUR- and GH3-proteins, whereas inhibition of cGMP biosynthesis had reversal effects. Furthermore, application of the cGMP analog enhanced auxin-induced degradation of Aux/IAA protein modulated by the SCF^TIR1^ ubiquitin-proteasome pathway. These results strongly support the concept that the stimulating role of NO and H_2_O_2_ on AR formation is partially mediated by a cGMP-dependent stimulation of auxin-induced degradation of Aux/IAA repressors (Figure 2). In addition, NO may provide a crossroad to other hormones such as ethylene, cytokinins, jasmonic acid, abscisic acid, salicylic acid, and gibberellins (Freschi, 2013). Considering NO production and removal mechanisms in plants (Freschi, 2013), an interesting question for the future is, whether auxin-mediated AR formation in cuttings can be enhanced by general stimulation of NO production, for example via enhancing the level of putative NO donors such as nitrate or arginine, by enhancing the activity of key enzymes of NO release such as nitrate reductase activity, or by reducing components of the NO removal machinery such as non-symbiotic hemoglobins. Lee et al. (2014) recently showed that water-logging-induced NO accumulation in roots of rape seedlings is the outcome of adjustment of both nitrate reductase activity and hemoglobin production.

KEY CONCEPT 6 **Auxin action on AR formation in cuttings involves NO- and H**_2_**O**_2_**-mediated signaling**.

Transcription factors (TFs) of the GRAS family such as SCARECROW (SCR) and SHORTROOT (SHR), of the AINTEGUMENTA-LIKE (AIL) family belonging to the APETALA 2/ETHYLENE RESPONSE FACTOR (AP2/ERF) domain transcriptional regulators, and WUSCHEL-related HOMEOBOX (WOX) proteins have important control functions during primary and lateral root development, linking auxin-signaling with cell specification and patterning and are also involved in feed-back regulation of local auxin homeostasis (Benjamins and Scheres, 2008; Ding and Friml, 2010; Horstman et al., 2014). There is increasing support in literature that GRAS TFs have also important control functions in AR formation in cuttings particularly in the context of maturation of woody plants, where they probably mediate the auxin control of cell fate in a phase- and cell-type-dependent manner and participate in auxin distribution (Sanchez et al., 2007; Vielba et al., 2011; Abarca et al., 2014). Recent studies further indicate that genes of the AP2/ERF family, which may also include certain ERFs mentioned above, have important functions in AR formation in cuttings. Rigal et al. (2012) showed that in poplar cuttings expression of the AIL-gene *PtAIL1* was enhanced during differentiation of root primordia and that the modified expression of this gene controlled the intensity of AR formation. The expression of one other poplar gene of the AP2/ERF family, *PtaERF003*, was induced by auxin and controlled the intensity of AR formation in cuttings appearing to act as a broad regulator of growth rather than to master root development (Trupiano et al., 2013). Recently it was shown by Xu et al. (2015), that also TFs of the WOX-type have regulatory functions in AR formation in poplar. Constitutive overexpression of either *PeWOX11a* or *PeWOX11b* strongly accelerated AR formation in poplar cuttings, increased the number of ARs and further induced ectopic roots in the aerial parts of plants. Considering further that *WOX5* is induced by auxin (Gonzali et al., 2005) and acts downstream of auxin-distribution in primary roots of *Arabidopsis* (Ding and Friml, 2010), the results altogether point toward an involvement of GRAS, AP2/ERF and WOX-type TFs in auxin-induced AR formation in cuttings (**Key Concept 7**, Figure 2).

KEY CONCEPT 7 **Auxin action on AR formation in cuttings involves TFs of the GRAS, AP2/ERF and WOX family** linking auxin signaling with cell fate determination and specification.

Until today no information is available about the functioning networks of auxin and TFs in cuttings. However, recent studies by Della Rovere et al. (2013, 2015) on isolated TCLs and intact hypocotyls of *Arabidopsis* provide interesting insights into the regulation of cell fate and specification during AR formation. Whereas, in intact hypocotyls ARs are initiated in the pericycle, in TCLs, i.e., stem inflorescence tissues external to the vascular system, ARs are initiated in the endodermis (Falasca et al., 2004). In primary and lateral roots, a group of less mitotically active cells, the quiescent Center (QC) is essential for maintaining undifferentiated cells in the apical meristem (Petricka et al., 2012). Localization of expression of a QC marker gene and of genes for candidate TFs and for auxin transporters by using promotor-reporter constructs together with immunolocalization of cytokinins under various auxin/cytokinin treatments revealed that independent of the founder cells, the QC is also established in the ARs (Della Rovere et al., 2013). The results indicate, that an auxin maximum determined by the coordinated activity of PIN1 and LAX3, an auxin-inducible influx carrier, confines WOX5 expression to the AR primordium tip, thereby positioning the QC. Responses to auxin application indicate that high auxin level promoted a PIN1-mediated lateral efflux from the vasculature toward the founder cells. During the later phase, *LAX3* was particularly expressed in the cells adjacent to the protruding AR primordium which points toward a function of this influx transporter in AR emergence. The results further indicate that cytokinins fine-tune the canalization and maximization of auxin via negatively affecting *PIN1* and *LAX3* expression. A following study on the roles of SHR and SCR TFs and the auxin influx carrier AUX1 completed the picture (Della Rovere et al., 2015). Whereas, null mutants of *SHR, SCR* or *AUX1* showed reduced AR formation, they exhibited a stimulation of xylogenesis indicating that both processes are regulated by these factors in a competitive manner. In wildtype plants, increased *SCR* expression started in the founder cells of ARs and continued to be present in primordia and elongating ARs. Considering its earliness and location of expression, Della Rovere et al. (2015) suggested *AUX1* as important factor related to the priming activity of SHR and SCR controlling rhizogenesis vs. xylogenesis. As a whole, these results support the conception that in cuttings auxin acts also as an initial signal to trigger a self-regulatory process of auxin canalization and maximization toward responding founder cells involving regulation of TFs of the GRAS, AP2/ERF and WOX-type (**Key Concepts 3, 7**, Figure 2).

In carnation cuttings, genes encoding microtubules (MTs) and MT-associated proteins were differentially expressed during AR formation, with a transient down-regulation during the early induction phase (termed as dedifferentiation phase) and up-regulation thereafter (Villacorta-Martin et al., 2015). MTs play essential roles in cell division and cell elongation and there is evidence supporting a function in organogenesis (Wasteneys and Fujita, 2006; Landrein and Hamant, 2013). A recent study on *E. grandis* indicated a particular role of MTs in auxin-induced AR formation in cuttings (Abu-Abied et al., 2014; **Key Concept 8**, Figure 2). Thus, time course analysis of 15,000 transcripts was used to detect candidate genes involved in the contrasting rooting capacity of juvenile (high) vs. mature (low) cuttings. Validation of candidate genes by the Nanostring method revealed that only juvenile cuttings responded to auxin application with enhanced expression of *PIN3* and of *IAA19*, an Aux/IAA repressor. However, the most pronounced difference was found in the expression of 42 transcripts annotated as coding for particular MT-associated proteins at 6–9 dpe, when the root primordia developed. The functional role of microtubule remodeling was supported by subtle perturbations of MTs using a drug, which partially rescued AR formation in mature cuttings. The authors proposed, that a coordinated developmental and auxin dependent remodeling of MTs contributes to the shift from cell division to cell differentiation, and further strengthened this concept by functional analysis in *Arabidopsis*. Auxin-induced AR formation in cut etiolated hypocotyls of the temperature-sensitive mutants *mor1-1* and *rid5*, in which the MT-associated protein MOR1 is mutated, was inhibited at the restrictive temperature of 29°C when compared with the wild-types (Abu-Abied et al., 2015a). The mutants produced amorphous clusters

KEY CONCEPT 8 **Auxin action on AR formation in cuttings involves microtubule and cell wall remodeling**.

of cells, instead of dome-like primordia seen in in wild-type plants. Detailed analysis of the organization of MTs, of cell wall properties, of auxin reception and PIN1 location also under the influence of chemical inhibitors of MT polymerization and cellulose synthesis supported the concept that generation of local maxima of auxin and its crosstalk between MTs and cell wall properties are important for the shift from cell division to cell differentiation during AR formation in *Arabidopsis* (Abu-Abied et al., 2015a). In dependence on auxin application, mutations in the MT-severing protein KATANIN reduced the rate and number of AR primordia and altered the expression of genes encoding MT-associated proteins along AR formation (Abu-Abied et al., 2015a,b). Further considering that MTs are sensitive to mechanical signals, the authors suggested that mechanical perception is part of the ARs differentiation program and that fine-tuning of MTs behavior is important for coordinated organ differentiation. According to this view, in the bottom part of cuttings of *Robinia*, expression levels of MT-related proteins increased during auxin-induced formation of AR primordia and elongation of ARs (Zhang et al., 2015). Confirming findings of Brinker et al. (2004) on auxin-induced AR formation in pine hypocotyls, recent studies involving RNA-seq analysis further underlined the important role of cell wall reorganization during AR formation (**Key Concept 8**, Figure 2). In cuttings of one carnation cultivar, 51 genes of the group “cell wall organization and biogenesis” showed a significant regulation during AR formation. Expression of most of these genes peaked at 6 hAP, before new meristematic cells were observed (Villacorta-Martin et al., 2015). Wei et al. (2014) showed that auxin application to cuttings of *Camellia* strongly modified the expression at 1 dpe of 40 genes related to cell wall weakening and modification. Recently Lewis et al. (2013) provided support for the functional role of enzymes controlling cell wall remodeling during lateral root development in *Arabidopsis* by use of insertion mutants.

## Conclusion and outlook

Based on the summarized literature, we propose a model on the regulation and involvement of auxin, ethylene and jasmonate homeostasis, signaling and function in AR formation in cuttings (Figure 2). Wounding, isolation from the donor plant and auxin application induce early transient accumulation of IAA and JA, early upregulation of ethylene biosynthesis and signaling and modifies local auxin biosynthesis and mobilization. Isolation-induced depletion of water and minerals in the rooting zone may additionally stimulate ethylene biosynthesis. The early PAT-dependent accumulation of IAA in the rooting zone acts as a trigger to initiate changes in auxin transport, signaling and function in particular responding cells. These changes initiate self-regulatory auxin canalization toward and maximization in particular cells, thereby starting the program of AR formation. This is followed by decrease in IAA as a result of auxin catabolism (function of peroxidases) and enhanced conjugation (GH3 proteins) supporting the differentiation of primordia during the root formation phase. Fine-tuned expression of TIR1/AFB complex components, of AUX/IAA proteins and of TPL are involved to guide the tissues through the different phases of AR formation. NO and H_2_O_2_ accumulation stimulated by wounding and auxin act as mediators of auxin signaling, probably via modification of the TIR1/AFB-Aux/IAA-ARF interaction, involving cGMP and MAPKs cascades. The function of auxin involves a phase- and cell-type specific regulation of TFs of the GRAS, AP2/ERF and WOX families. Aux/IAA, TPL, GH3- and SAUR-proteins, ARFs, ERFs, TFs of the GRAS, AP2/ERF and WOX-family, and NO provide important links to other hormones. The implementation of the auxin signal at cellular and tissue level involves the control of cell cycle, microtubule and cell wall remodeling and readjustment of carbohydrate metabolism toward establishment of a new sink. Stress- and auxin-induced stimulation of ethylene biosynthesis and perception and early transient accumulation of JA stimulate the process of AR formation, while probably both pathways are linked to auxin homeostasis and signaling. Depending on the plant species, JA may also act via activation of invertases.

New methods for broad evaluation of the transcriptome using e.g., RNA-seq technology are currently applied and will provide new candidate genes also in non-model plants. Such transcriptome data should be combined with proteomic, metabolic and cytological data, to identify regulative components and describe processes on all levels during AR formation (**Key Concept 9**). In order to move further from description to functional analyses of single genes or entire processes, the expression of particular genes must be modulated by the use of mutants, RNAi technology and by overexpression. Inducible or tissue-specific promotors will be necessary to assign functions to particular phases and tissues.

KEY CONCEPT 9 **Understanding of hormonal control in excision-induced AR formation needs to (1) further explore the regulation** at the levels of transcriptome, proteome and metabolome **(2) assign the function of components to tissue and cell level** and **(3) consider the whole cutting as a functional system** of distinct multi-functional physiological units responding to the environment and determining the rooting response.

Finally, integration of the rooting zone into the whole resource distribution and signaling system of the cutting will be important to better understand AR formation in the context of modern breeding and propagation technology with its diverse genetic and environmental cues. Depending on the type of the cutting, different cutting parts should be considered as functional units (Figure 2) specifically responding to environmental factors and limiting AR formation in the rooting zone via allocation or withdraw of important signals and resources. A better understanding of the control and function of the intrinsic hormonal networks in this developmental process would enhance our knowledge of plant plasticity and unravel starting points for improvement of plant propagation and breeding.

## Author contributions

UD wrote the paper. PF and MRH edited the manuscript.

## Funding

Our research providing the basis for this review was funded by the Pakt für Forschung und Innovation of the Leibniz-Gemeinschaft and by the Deutsche Forschungsgemeinschaft (DR 411/2-1) and further supported by the States of Brandenburg and Saxony-Anhalt, the Free State of Thuringia and the Federal Republic of Germany.

### Conflict of interest statement

The authors declare that the research was conducted in the absence of any commercial or financial relationships that could be construed as a potential conflict of interest.

## Abbreviations

ABC: ATP-binding cassette
ACC: aminocyclopropane
ACO: ACC oxidase
ACS: ACC synthase
AIL: AINTEGUMENTA-like
AP2/ERF: APETALA2/ETHYLENE RESPONSE FACTOR
AR: adventitious root
ARF: auxin response factor
AUX: auxin permease
cGMP: calcium cyclic guanosine monophosphate
dpe: days post excision
ERF: ethylene responsive transcription factor
hpe: hours post excision
hAP: hours after planting
IAA: indole-3-acetic acid
IAA-AAH: IAA-amino acid hydrolase
JA: jasmonic acid
MAPK: mitogen-activated protein kinase
LAX: like AUX
MDR/PGP: multidrug resistance/P-glycoprotein
MT: microtubule
NPA: naphthylphthalamic acid
PAT: polar auxin transport
PIN: pin-formed
RNA-seq: RNA sequencing
SAUR: SMALL AUXIN UP RNA
SCR: SCARECROW
SHR: SHORTROOT
TCL: thin cell layer
TF: transcription factor
TIR1/AFB: transport inhibitor response/auxin-signaling F-box
TPL: TOPLESS
QC: quiescent center
WOX: WUSCHEL-related homeobox.
